# Shewanella putrefaciens: A Rare Cause of Refractory Peritonsillar Abscess in an Immunocompetent Adult

**DOI:** 10.7759/cureus.107635

**Published:** 2026-04-24

**Authors:** Sahar Rochd, Almahdi Afroukh, Abderrahim Oumloul, Taoufik Ben Houmich, Asmae Lamrani Hanchi, Nabila Soraa

**Affiliations:** 1 Laboratory of Microbiology, Faculty of Medicine and Pharmacy of Marrakech-Cadi Ayyad University, Mohammed VI University Hospital, Marrakech, MAR; 2 Department of Microbiology, Faculty of Medicine and Pharmacy of Marrakech-Cadi Ayyad University, Mohammed VI University Hospital, Marrakech, MAR; 3 Department of Microbiology, Arrazi Hospital, Mohammed VI University Hospital, Marrakech, MAR

**Keywords:** maldi-tof ms, peritonsillar abscess, rare pathogen, refractory infection, shewanella putrefaciens

## Abstract

*Shewanella* species are motile, oxidase-positive, non-fermenting Gram-negative bacilli widely distributed in marine and aquatic environments. Although primarily environmental organisms, *Shewanella* spp. are rare human pathogens, most often associated with skin and soft tissue infections. Involvement of the head and neck region is exceptional.

We report the case of a 28-year-old immunocompetent male with no history of seawater exposure who presented with a one-week history of odynophagia, fever, trismus, and dysphagia. Clinical examination revealed a unilateral peritonsillar abscess. Needle aspiration yielded hemopurulent material. Direct microscopy showed numerous neutrophils and Gram-negative bacilli. Culture on standard media grew monomorphic, non-lactose-fermenting colonies. Identification by matrix-assisted laser desorption/ionization time-of-flight mass spectrometry (MALDI-TOF MS) (Bruker Daltonics, Bremen, Germany) confirmed *Shewanella putrefaciens* with a high-confidence score.

Antimicrobial susceptibility testing demonstrated resistance to penicillins, β-lactam/β-lactamase inhibitor combinations, and cephalosporins, with preserved susceptibility to fluoroquinolones, aminoglycosides, and carbapenems. Empiric therapy with amoxicillin-clavulanate was ineffective, whereas targeted treatment with levofloxacin resulted in complete clinical recovery.

This case highlights *S. putrefaciens* as an uncommon but clinically relevant cause of refractory peritonsillar abscess and underscores the importance of accurate microbiological identification, particularly in patients not responding to standard empiric therapy.

## Introduction

*Shewanella* species are motile, oxidase-positive, non-fermenting Gram-negative bacilli that are facultatively anaerobic and capable of producing hydrogen sulfide on triple sugar iron agar [1,2]. They are widely distributed in marine and aquatic environments and are primarily considered environmental organisms. Ecologically, they play a beneficial role in nutrient cycling and bioremediation, particularly by reducing heavy metals, thereby contributing to environmental balance [3].

Human infections caused by *Shewanella* spp. are uncommon. They are most frequently associated with skin and soft tissue infections, otitis, and occasionally bacteremia, often in patients with underlying comorbidities or a history of seawater exposure [4]. Among the genus, *Shewanella putrefaciens* and *Shewanella algae* are the species most frequently implicated in human disease. Accurate differentiation between these two species remains challenging using conventional phenotypic methods [2,4].

Head and neck infections due to *Shewanella* spp. are exceptionally rare, with only a limited number of cases reported worldwide [2,5]. In this report, we describe a rare case of peritonsillar abscess caused by *S. putrefaciens* in an immunocompetent patient without known environmental exposure.

## Case presentation

A 28-year-old male with no significant medical history and no reported exposure to seawater presented to the Emergency Department with a one-week history of sore throat, odynophagia, dysphagia, trismus, and fever.

On physical examination, the patient was febrile (39°C) with a preserved general condition. Oropharyngeal examination revealed unilateral peritonsillar swelling with marked erythema and purulent exudate covering the enlarged tonsil. Ipsilateral tender cervical lymphadenopathy was noted. There were no signs of airway compromise. These findings were consistent with a peritonsillar abscess.

Laboratory investigations revealed an inflammatory syndrome characterized by elevated C-reactive protein (22.6 mg/L) and leukocytosis (11.93 × 10⁹/L) with neutrophil predominance. Hemoglobin concentration and platelet count were within normal limits. Renal and hepatic function tests were unremarkable.

Needle aspiration of the peritonsillar swelling was performed under local anesthesia, yielding hemopurulent material and confirming the diagnosis. Direct microscopic examination of the aspirate showed numerous neutrophils and Gram-negative bacilli.

**Figure 1:**
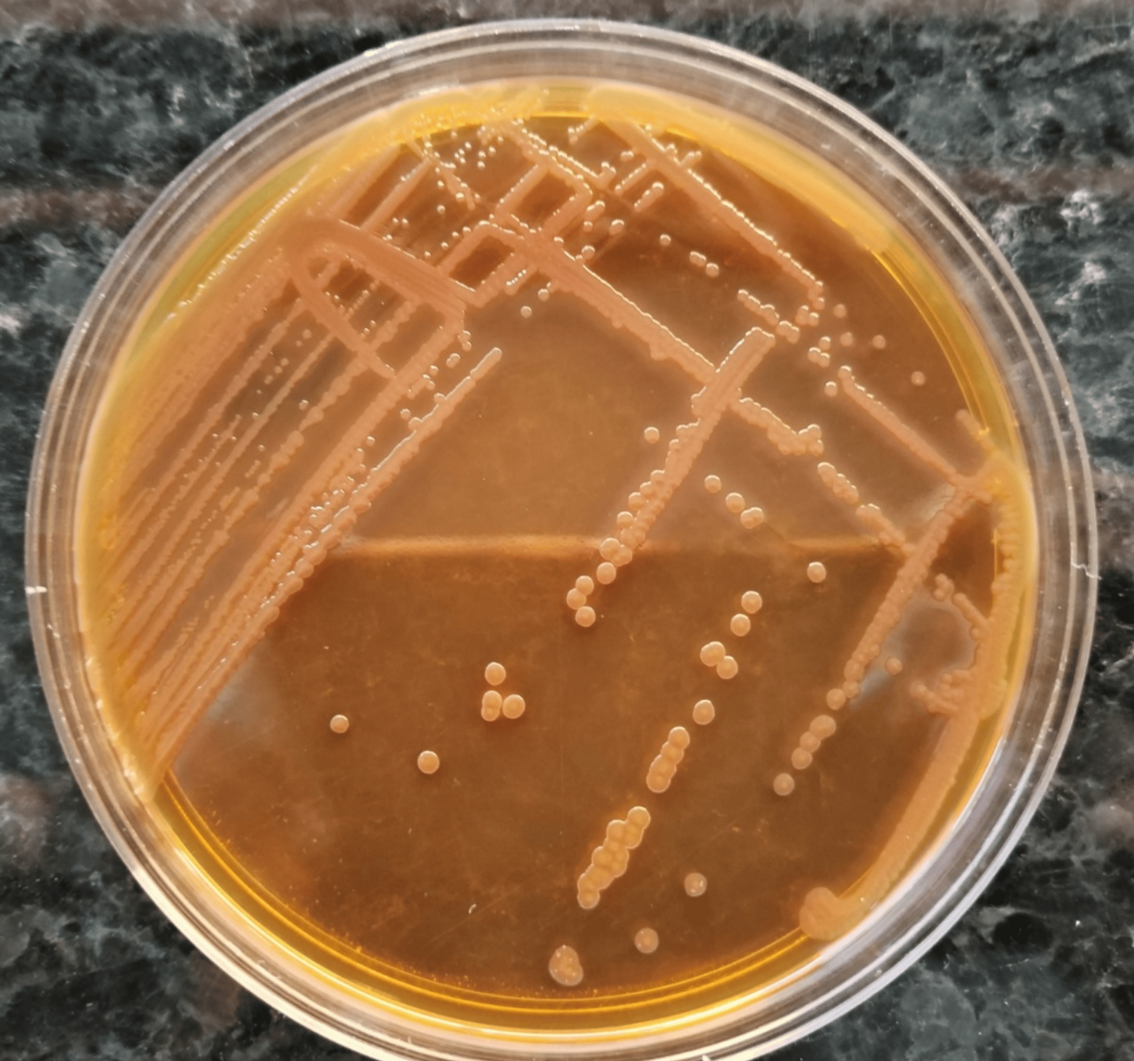
Young culture of Shewanella putrefaciens on blood agar after 24 hours of incubation

Culture was performed on chocolate agar, MacConkey agar, and Columbia agar with colistin and nalidixic acid, incubated at 37°C under appropriate atmospheric conditions. After 24 hours, cultures yielded monomorphic, non-lactose-fermenting colonies (Figure 1). Identification by matrix-assisted laser desorption/ionization time-of-flight mass spectrometry (MALDI-TOF MS) (Bruker Daltonics, Bremen, Germany) confirmed *S. putrefaciens* with a high-confidence score.

Antimicrobial susceptibility testing was performed using the disk diffusion method on Mueller-Hinton agar, according to the Comité de l’Antibiogramme de la Société Française de Microbiologie/European Committee on Antimicrobial Susceptibility Testing (CA-SFM/EUCAST) recommendations [6], with extrapolated breakpoints for non-fermenting Gram-negative bacilli. The isolate was resistant to penicillins, β-lactam/β-lactamase inhibitor combinations, and cephalosporins and susceptible to fluoroquinolones, aminoglycosides, and carbapenems (Table 1).

**Table 1:** Antimicrobial susceptibility profile of Shewanella putrefaciens Note: S = susceptible; R = resistant.

Antibiotic	Inhibition zone diameter (mm)	Result
Amoxicillin-Clavulanate	15.5	R
Piperacillin-Tazobactam	14	R
Ceftriaxone	13	R
Ceftazidime	≤ 6	R
Cefepime	14	R
Erythromycin	13.5	R
Clindamycin	≤ 6	R
Trimethoprim-Sulfamethoxazole	≤ 6	R
Imipenem	26	S
Ertapenem	14	-
Gentamicin	23.2	S
Amikacin	15.7	S
Ciprofloxacin	28.9	S
Levofloxacin	30	S

Empiric treatment with amoxicillin-clavulanate was initiated but resulted in no clinical improvement. Based on susceptibility results, treatment was switched to levofloxacin, leading to complete clinical recovery after 10 days.

## Discussion

Human infections caused by *Shewanella* spp. remain rare and are predominantly associated with skin and soft-tissue infections, otitis, and bacteremia, often in patients with underlying comorbidities or a history of seawater exposure. Head and neck infections due to *Shewanella* species are exceptional, with only a limited number of cases reported worldwide [7-9]. To our knowledge, no previous cases of peritonsillar abscess caused by *S. putrefaciens* have been reported in Morocco.

Accurate identification of *Shewanella* species is challenging because of the close phenotypic similarity between *S. putrefaciens* and *S. algae*. Conventional biochemical methods may lead to misidentification, which can delay appropriate antimicrobial therapy. In this context, MALDI-TOF MS has emerged as a rapid and reliable tool for species-level identification and is now considered a best-practice component of the diagnostic workflow for uncommon Gram-negative pathogens [10,11].

The pathogenic mechanisms of *S. putrefaciens* are not fully elucidated. However, several virulence-associated traits have been described, including biofilm formation, production of extracellular enzymes, and the ability to survive in low-oxygen or anaerobic conditions [2,5]. These characteristics may contribute to persistence in deep-seated infections and failure of empiric antimicrobial therapy [1,2]. In the present case, the isolation of *S. putrefaciens* in monomorphic culture, together with abundant neutrophils and Gram-negative bacilli observed on direct microscopy, supports its role as the primary pathogen rather than a contaminant.

Antimicrobial susceptibility patterns reported for *Shewanella* spp. are variable but commonly include resistance to penicillins, β-lactam/β-lactamase inhibitor combinations, and cephalosporins, while fluoroquinolones, aminoglycosides, and carbapenems generally retain activity [1,12]. The resistance profile observed in this case is consistent with previous reports and is likely related to the production of chromosomal β-lactamases [13,14]. The failure of empiric amoxicillin-clavulanate therapy in this patient underscores the importance of reconsidering antimicrobial coverage in patients with peritonsillar abscesses who do not respond to standard empiric treatment [15,16].

Although *Shewanella* infections are often associated with environmental exposure, particularly seawater, no such exposure was identified in this case. This observation suggests that alternative routes of acquisition may exist and highlights the need to consider *Shewanella* spp., even in the absence of classical risk factors [5].

Limitations

This report describes a single clinical case, which limits the generalizability of the findings. Although species identification was achieved using MALDI-TOF MS with a high-confidence score, molecular confirmation (e.g., 16S rRNA gene sequencing) was not performed, and species-level misidentification cannot be entirely excluded. Antimicrobial susceptibility testing relied on disk diffusion with extrapolated CASFM/EUCAST breakpoints, due to the absence of species-specific interpretative criteria for *Shewanella* spp., which may affect susceptibility interpretation. Finally, the source of infection could not be identified, as no environmental investigation was conducted.

## Conclusions

*S. putrefaciens* should be considered a rare but clinically relevant pathogen in refractory peritonsillar abscesses. This case highlights the importance of accurate microbiological identification using MALDI-TOF MS to guide targeted antimicrobial therapy and avoid prolonged clinical failure with empiric treatment. Increased awareness of uncommon Gram-negative pathogens may improve diagnostic accuracy and patient outcomes in head and neck infections.
